# Outstanding animal studies in allergy II. From atopic barrier and microbiome to allergen-specific immunotherapy

**DOI:** 10.1097/ACI.0000000000000364

**Published:** 2017-04-25

**Authors:** Erika Jensen-Jarolim, Isabella Pali-Schöll, Franziska Roth-Walter

**Affiliations:** a Institute of Pathophysiology and Allergy Research, Center of Pathophysiology, Infectiology and Immunology; b The Interuniversity Messerli Research Institute, University of Veterinary Medicine Vienna, Medical University of Vienna, University of Vienna, Vienna, Austria

**Keywords:** allergen-specific immunotherapy, allergy, atopic dermatitis, barrier, diet, microbiome, mouse model

## Abstract

**Purpose of review:**

Animal studies published within the past 18 months were assessed, focusing on innate and specific immunomodulation, providing knowledge of high translational relevance for human atopic and allergic diseases.

**Recent findings:**

Allergic companion animals represent alternative models, but most studies were done in mice. Atopic dermatitis mouse models were refined by the utilization of cytokines like IL-23 and relevant skin allergens or enzymes. A novel IL-6 reporter mouse allows biomonitoring of inflammation. Both skin pH and the (transferable) microflora have a pivotal role in modulating the skin barrier. The microflora of the gastrointestinal mucosa maintains tolerance to dietary compounds and can be disturbed by antiacid drugs. A key mouse study evidenced that dust from Amish households, but not from Hutterites protected mice against asthma. In studies on subcutaneous and sublingual allergen-specific immunotherapy, much focus was given on delivery and adjuvants, using poly-lacto-co-glycolic particles, CpGs, probiotics or Vitamin D3. The epicutaneous and intralymphatic routes showed promising results in mice and horses in terms of prophylactic and therapeutic allergy treatment.

**Summary:**

In atopic dermatitis, food allergies and asthma, environmental factors, together with the resident microflora and barrier status, decide on sensitization versus tolerance. Also allergen-specific immunotherapy operates with immunomodulatory principles.

## INTRODUCTION

Failure in innate defense mechanisms (inborn or acquired barrier leakage of skin and mucosa and misbalanced microbiome) often is the first step toward atopic and allergic diseases. Animal models should help us mimicking these pathophysiological conditions and should allow proof-of-concept studies for the efficacy of novel therapies with a certain predictive value for humans. Many aspects of mouse models are suitable to picture the human condition in its extreme and were very successfully used also in current animal models of allergy and immunomodulation. Several immune parameters can be directly compared between animal models and humans; for instance, mice harbor natural killer T cells, dendritic cells (although lacking FcεRI in wild type mice) and regulatory T-cells (Tregs). However, mouse models are limited, e.g. because of the absence of the immunoglobulin G4 (IgG4) immunoglobulin class, which is a prominent hallmark of successful allergen immunotherapy and was recently suggested as a reliable marker for the compliance and adherence of patients to allergen-specific immunotharepy [1].

**FIGURE 1:**
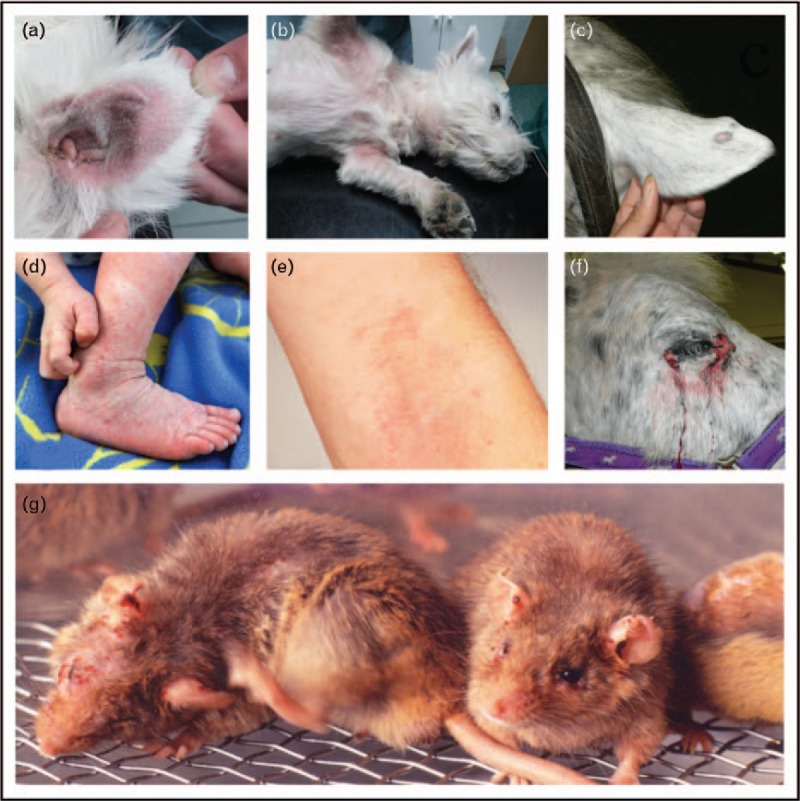
Typical atopic dermatitis lesions in domestic animals and humans versus NC/Tnd mice. Atopic lesions in a Maltese dog's ear (a) and subaxillary (b); itchy atopic dermatitis (neurodermitis) in a child (d) and in flexural site of a human adult (e); on ear and around the eye of a horse (c, f) (a–f from [3], reproduced with permission of Springer); (g) in an NC/Tnd mouse an inbred strain originating from NC/Nga, modeling all features of natural atopic dermatitis including barrier leakage and itchiness (by courtesy of Professor Hiroshi Matsuda, Tokyo University of Agriculture and Technology, Japan).

In comparison, other mammals, for instance canines, express four immunoglobulin subclasses and a similar immunoglobulin E (IgE)-receptor repertoire on the same cells like humans, including eosinophils and dendritic cells. Therefore, to overcome the limitations of murine models, atopic and allergic domestic animals could be investigated as natural models of disease with a potentially higher predictive value [2,3] (Fig. 1). 

**Box 1:**
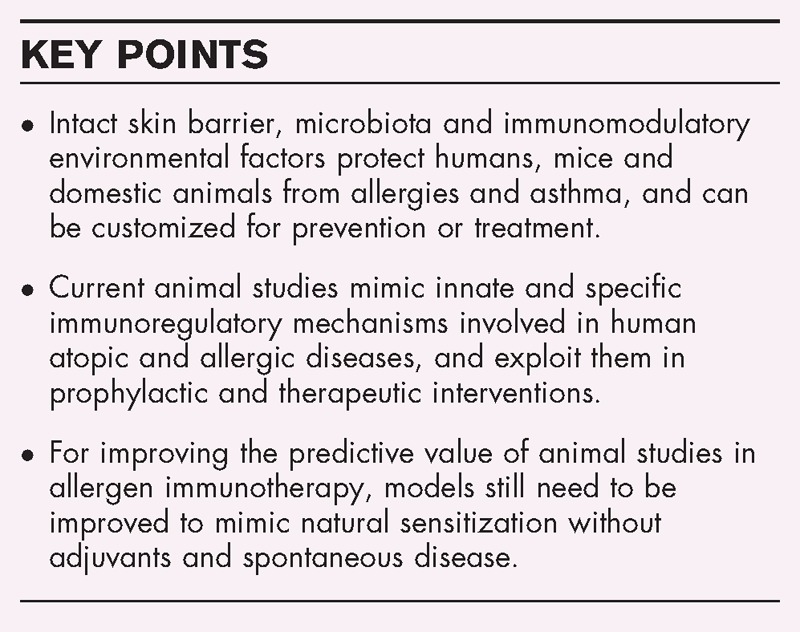
no caption available

It has to be emphasized that most of the generated knowledge discussed below derives from murine studies, but prompted key observations with respect to the power of prophylactic or therapeutic treatments, innate or specific immunomodulation, being translatable for humans.

## ATOPIC DERMATITIS MODELS

The barrier function of a healthy skin is characterized by intact intercellular junctions between keratinocytes fixed by a ceramide glue, being supported by acidic skin pH and an intact microflora that via epithelia is in constant dialogue with the cutaneous innate immune defense. The tolerance to skin commensals is established very early in neonatal life by a wave of Tregs, which invade the skin [4]. This was shown by engineered *Staphylococcus epidermidis* expressing a model T-cell antigen (model peptide antigen 2W linked to a fluorescent protein) applied to disrupted skin: only in 7 days old (but not in adult) C57Bl/6 mice, Foxp3^+^ Tregs increased within the antigen-specific CD4^+^ population in the skin-draining lymph nodes and skin.

In atopic dermatitis on the contrary, innate and specific immune defense arms accelerate acute toward chronic inflammation linked with a mixed Th1/Th17/Th2 immune response [5] (Fig. 1d, e). Disturbances in the skin and mucosal barrier are typically associated with a misbalanced microflora, which may be the ‘hen or the egg’ in this disease. As a result, atopic patients have a higher risk for developing allergies. Several animal models, mostly in mice, have been created or applied recently to mimic atopic dermatitis and to understand these interconnected problems.

### Immune Effects of the Microflora in Atopic Dermatitis

Although pathogenic skin bacteria via interleukin (IL)-23 strongly boost Th1/Th17 inflammation, other stimuli such as enzymes via protease-activated receptor (PAR)-2 and thymic stromal lymphopoietin (TSLP) trigger Th2 responses, involving basophil-derived IL-4 [6]. Both counteracting axes contribute to the mixed phenotype in human atopic dermatitis patients and could in fact be mimicked in mouse and canine models [7]. In a transcriptomic profiling study, however, IL-23-injected, NC/NgaTnd [8] (Fig. 1 g) and oxazolone-challenged mice had the highest homology to human disease, each reflecting different immune or barrier aspects [9^▪▪^].

Dogs’ spontaneous atopic dermatitis not only has inflammatory and prurigenic pathways like humans [10] (Fig. 1 a,b), but also a reduced microbiome diversity with a dominance of *Staphylococcus aureus* and *Corynebacterium* species, which correlate with barrier leakage [11]. Only gram-negative bacteria collected from healthy controls, but not from atopic dermatitis patients, were able to maintain barrier integrity and control of *Staphylococcus* when inoculated in MC903 atopic dermatitis mice [12^▪▪^], suggesting a ‘live-biotherapeutic approach’ for human patients.

The chronic aspects in atopic eczema are accompanied by IL-6 induction. This can now be monitored *in vivo* by bioluminescence imaging in the novel AhR-CA transgenic mouse model [13^▪^], allowing also transverse section image. In this lipopolysaccharid-induced luciferase reporter system, ‘WIM-6’ luminescence could be detected in each tissue, allowing the monitoring of progression of atopic dermatitis as well as treatment efficacy with dexamethason.

Environmental factors may also disturb the robustness of the skin. Alkalization of the skin of NC/Tnd mice induced epidermis kallikrein-5 mRNA and protein, an endogenous serine protease, resulting in PAR-2 activation, TSLP secretion and a cutaneous Th2 response, but could be blocked by PAR-2 antagonist ENMD-1068 [14]. As their phenotype could be counterbalanced by acidification, the study emphasizes the pivotal role of the physiological, slightly acidic cutaneous pH and translates into care recommendations for atopic dermatitis skin.

The exogenous enzyme papain, a prototypic cysteine protease, was used as surrogate for the house dust mite allergen Der p 1 for several studies. Enzymatically active papain induced immediate inflammation via intact skin *in vivo*, and it retained the sensitization potential when its enzymatic activity was inhibited [15]. On tape stripped skin, papain acted as adjuvant for another model allergen, ovalbumin [16], and primed specific respiratory hypersensitivity, mirroring the atopic march.

Interestingly, silver particles adjuvanted house dust mite proteins to become percutaneous allergens, thereby aggravating atopic dermatitis and activating mast cells [17].

Intrinsic barrier defects play a major role in the development of atopic dermatitis. A filaggrin mutant mouse on BALB/c background [18], like previously NC/Nga mice [19], developed spontaneous atopic dermatitis and compromised pulmonary function, thereby also mimicking the atopic march. Intriguingly, it could be healed by application of acidic skin cream [19].

Reduced expression of tight junction compound claudin-1 correlated with more severe atopic dermatitis in mice [20]. Being pronounced in infancy, symptoms improved with age, imitating the course of human atopic eczema.

Also, adherens junctions contribute to the regulation of the extracellular matrix: in a cadherin-11 knockout mouse model, cutaneous levels of elastin and collagen as well as mechanical properties were significantly reduced [21].

By concentrating on keratinocyte turnover, another useful atopic dermatitis model was developed: epidermis-restricted ablation of the proto-oncogene BRAF/RAF1 led to barrier defects and local and systemic IgE and Th2 inflammation; Janus kinase inhibition prevented disease onset [22].

In terms of potential novel therapies in atopic dermatitis, a proof-of-concept study in NC/Nga mice sensitized by papain revealed that Bacillus-derived poly-γ-glutamic acid-treatment induced invariant natural killer T cells driving basophils into apoptosis, thereby correcting the pronounced Th2 inflammation [6].

Astaxanthin, a xanthophyll carotenoid [23], and AM1030, a serotonin antagonist [24], showed promising anti-inflammatory effects, and targeted the itch in NC/Nga mice. Allergen-specific oral tolerance could be achieved in an atopic dermatitis mouse model epicutaneously sensitized to ovalbumin, with practical implications in human atopic dermatitis patients suffering from exacerbations due to the intake of dietary allergens [25]. A study in atopic dogs, which represent a relevant model for human atopic dermatitis [3], demonstrated that a chemokine receptor-4 antagonist prevented homing of lymphocytes into the lesional skin [26].

## THE MICROBIOME: INNATE IMMUNOMODULATION IN FOOD ALLERGY AND ASTHMA MODELS

The influence of diet on the microbiome and subsequent food allergy development is today well accepted [27]. For instance, in a house dust mite-mouse model, a high-fiber diet increased Bacteroidetes, especially high-acetate-producing *Bacteroides acidifaciens*, which produce high levels of short-chain fatty acids like acetate in serum and feces [28]. These short-chain fatty acids inhibited histone deacetylase-C9 and prevented asthma development via Tregs. These findings were even true in offspring when mothers were on high-fiber diet during pregnancy. Asthma-preventive effects were mediated *in utero* predominantly by acetate (not other short-chain fatty acids), and independent of transfer of specific microbiota.

### Medications and Microbiota

There is also a link between the gastric digestion capability, the establishment of a specific microbiota and the development of food allergy: mice orally treated with ovalbumin concomitantly with antiacid drugs which impair the gastric peptic digestion differed in the fecal microbiome and subsequently developed allergic and even anaphylaxis symptoms [29].

### Environment and Housing

For the microbiological composition, environmental aspects also play an important role. Laboratory mice live under abnormal specific pathogen free (SPF) conditions and lack differentiated memory CD8^+^ T-cell subsets and mucosal memory T cells. When SPF animals were cohoused with pet store mice [30^▪^], their resistance to infection and T-cell differentiation upon de-novo viral infection increased, making ‘dirty’ mice valuable models for investigating, immune function and treatments in transplantation, allergy, autoimmunity and vaccination in context with the hygiene hypothesis [31].

Allergy-preventive effects are not the same on every farm: Amish folks were better protected against asthma than Hutterite people, implicating that the different living conditions are decisive in the two populations with almost identical genetic background [32^▪▪^]. Higher endotoxin levels in the house dust of Amish were made responsible for the observed protective effects. In accordance, aqueous house dust samples given intranasally concomitant with allergen sensitization protected mice during allergen challenge in an ovalbumin-asthma mouse model, whereas MyD88/Trif (innate immunity signaling molecules)-deficient mice were not protected.

### Probiotics

*Lactobacillus gasseri* OLL2809 (LG2809) fed in a DO11.10-mouse model (carrying a T cell receptor transgene, Tg (DO11.10) reactive to ovalbumin peptide) together with ovalbumin resulted in induction of oral tolerance, shown as decreased T-cell proliferation and IL-2 levels but increased IL-10 from spleen cells and more plasmacytoid dendritic cells in the lamina propria [33]. In another ovalbumin-food allergy BALB/c mouse model [34], the administration of probiotic *Bifidobacterium longum* KACC 91563, but not *Enterococcus faecalis* KACC 91532, via the intragastric route or in the food, applied concomitantly with sensitization, alleviated food allergy symptoms (diarrhea). This was rather due to apoptosis induction in mast cells but not in T cells, B cells or eosinophils, than by production of acetate.

To study immunomodulatory effects, human microbiota-associated mice are widely used, wherein a human fecal microbiome was established in germ-free mice through microbiota transplantation [35,36].

## IMMUNOMODULATION BY ALLERGEN-SPECIFIC IMMUNOTHERAPY

Barrier integrity and immune maintenance described above relate to allergen-independent mechanisms. In contrast, allergen-specific immunotherapy so far is the only causative, allergen-specific treatment of IgE-mediated allergic disorders. The mechanisms involve the induction of Tregs and blocking IgG4 antibodies, altogether resulting in immunomodulation combating the allergic Th2 bias. In general, repeated administration of increasing amounts of allergens results in a state of clinical hypo-responsiveness to the respective allergen. Conventional treatment usually employs the subcutaneous or the sublingual route. Mouse models of allergen immunotherapy are challenging for the following reasons: allergy mouse models rely on allergen application in context with Th2 adjuvants (often by nonphysiological routes) and thereby induce robust disease that is difficult to modulate; symptomatic allergy in mice is, unlike in humans, associated with IgE and IgG1; mice express Tregs, but do not harbor IgG4 antibodies which is an important biomarker in human allergen immunotherapy [1]. This makes comparative trials in domestic animal patients with spontaneous allergies and 4 IgG subclasses an attractive alternative approach.

### Immune Regulation by Sublingual Immunotherapy

In humans, the efficacy of sublingual immunotherapy depends on the sensitization level of the individual and the dose administered during therapy. Indeed, highly sensitized mice required higher sublingual immunotherapy (SLIT) dose than less sensitized mice to improve airway hyperresponsiveness [37]. Moreover, upon SLIT treatment, a lower proportion of CD4-CD8 γδ cells and a higher frequency of CD8^+^CD25^+^IFNγ^+^ T cells accompanied by reduced inflammatory responses in the lung were observed [38]. The efficacy of SLIT may also depend on Vitamin D3 administration that potentiated subcutaneous allergen immunotherapy in mice by accumulating Tregs in the lung in an allergen-driven manner [39]. Interestingly, the smoke compound acrolein, in a completely antigen-independent manner, had a similar effect as it prevented allergic sensitization by promoting suppressive T cells via aryl hydrocarbon receptor activation [40].

### Encapsulation for Safety in Subcutaneous Application

Focus was given on encapsulation procedures of allergy vaccines to enhance not only the immunogenicity, but also to confer safety. Strontium-doped hydroxyapatite porous spheres have been used in subcutaneous immunotherapy trials in mice to reduce side-effects, resulting in reduced symptoms after nasal challenge despite the higher percentage of eosinophils found in the bronchial lavage samples [41].

### Alternative route: oral

Although subcutaneous allergen immunotherapy is extensively used for some respiratory and venom allergies, it is unsuitable for food allergens because of the high risk of adverse reactions. In a study using the oral route as a safer alternative, ovalbumin was applied in conjunction with the probiotic *Clostridium butyricum* to reduce symptoms in a food allergic murine model [42^▪^]. The in-vitro data suggested that by addition of *C. butyricum,* ovalbumin-specific regulatory B cells were generated and the plasmacytic differentiation of B cells was inhibited. However, oral immunotherapy usually does not confer a sustained protection.

### Alternative routes: epicutaneous

In the pioneering experiments of epicutaneous immunotherapy without the use of adjuvant or tape stripping, sustained protection against anaphylaxis was induced by de-novo generation of latency-associated peptide^+^ Tregs, which was not achieved by the oral route in allergic mice. Tregs did not function by suppressing IgE antibodies, but instead directly suppressed mast cell activation, leading to sustained clinical protection against food-induced anaphylaxis [43^▪▪^]. Another study aimed to improve epidermal delivery of allergen using a microfractional laser to generate an array of self-renewable microchannels in the skin [44]. Epidermal application of allergens containing CpG and VitaminD3 turned out to be superior to the same treatment by the subcutaneous route, and resulted in a significant reduction of airway wall thickness, requiring a lower number of treatments. Similarly, in a murine atopic dermatitis model, epicutanous application of ovalbumin in conjunction with CpG was effective for treatment by promoting a strong Th1-mediated immune response leading to a decrease in antigen-specific IgE and increase in IgG2a antibodies [45].

### Alternative routes: intravenous

Allergen-specific immunotherapy by the intravenous route was considered safe and effective when using allergen entrapped into biodegrable poly-lacto-glycolic (PLG) particles, whereas surface-conjugation of the allergen to PLG particles showed less efficacy [46^▪▪^].

### Alternative routes: intralymphatic

In studies using the novel hCD2-DsRedxProx1-green fluorescent protein mice, featuring red T cells and green lymphatics, intravital microscopy depicted that lymphocytes crawl intralymphatically from inflamed tissue to lymphnodes [47^▪▪^].

Also the antigen itself was cargoed to lymph nodes by plasmocytoic dendritic cells, which resulted in tolerogenic effects [48].

In a proof-of-concept study for allergen-specific immunotherapy, intralymphatic application with ovalbumin adjuvanted with bacterial flagellin, a Toll-like receptor 5 agonist, was advantageous over the intranasal and sublingual applications in reducing symptoms in ovalbumin-sensitized mice [49^▪^].

Also for the intralymphatic allergy vaccines, comparative studies in veterinary patients are useful and were proposed for the treatment of canine atopic dermatitis [50]. Apart from atopy (Fig. 1c, f), insect bite hypersensitivity is a common problem in horses, which could be prevented by intralymphatic injections with recombinant insect allergens, adjuvanted with monophosphoryl lipid A (MPL) or aluminium hydroxide [51].

## CONCLUSION

Innovative efforts were undertaken in models for atopic dermatitis by exploiting mediators previously correlated with atopic dermatitis, such as IL-23 [8], or using IL-6 for biomonitoring atopic dermatitis [12^▪▪^]. Notably, it was demonstrated that the atopic dermatitis microbiota was transferable and, vice versa, inoculation of mouse skin with healthy microbiota corrected the disease [11]. Novel mouse models helped to understand the significance of the skin barrier, enzymatic allergens [14] and physicochemical factors like skin pH. Also in atopic dermatitis models, the great influence of the microbiota was apparent. Animal studies provided evidence that the diet and gastric digestive functions affect colonization of microbiota [29] and correlate with food allergy and asthma, or protect against it. The induction or maintenance of tolerance to allergens could be supported by probiotics [42^▪^], but even more by immunostimulatory environmental compounds [32^▪▪^]. Germ-free mice transplanted with human microbiome [35] are valid models, but have limitations, such as the incorrect host specificity for human microbes, the greater microbial colonization of the small intestine in mice and disregard of host-specific coevolutionary factors such as resistance and nutrient utilization [52].

A plethora of animal models for specific allergen immunotherapy tried optimizing the application mode and route of allergen, by entrapment in tolerogenic PLG particles [46^▪▪^], or application concomitantly with CpG [45], probiotics or Vitamin D3 (Table 1) [37–39,41,42^▪^,^43^^▪▪^,^44^,^45^,^46^^▪▪^,49^▪^,^51^]. In particular, epicutaneous application has achieved promising results without adjuvants [43^▪▪^]. On the basis of therapeutic mouse studies [49^▪^] and preventive approaches in horses [51], the frequency of usage of the intralymphatic route is expected to increase in the near future.

## Acknowledgements

No funding was obtained from National Institutes of Health (NIH), Wellcome Trust and Howard Hughes Medical Institute (HHMI).

### Financial support and sponsorship

The work to this article was supported by grant SFB F4606-B28 of the Austrian Science Fund FWF to EJJ.

### Conflicts of interest

The other authors declare no conflicts of interest.

## REFERENCES AND RECOMMENDED READING

Papers of particular interest, published within the annual period of review, have been highlighted as:

▪ of special interest
▪▪ of outstanding interest

## Figures and Tables

**Table 1:** Allergen immunotherapy models

Allergen	Sensitization	Therapy	Challenge	References
Der p 2	2 × 1 μg rDer p 2 + 2 mg Alum i.p.	Chemically modified monomeric allergoid of Derp2, d2-OID or d2-OID + VitD3	1 × 1 μg rDer p 2 i.p. and 5 × 1% HDM	(Petrarca C *et al.*) [39]
HDM	3, 5 or 7 weeks: 5 × 25 μg Der f extract i.n.	SLIT 2 weeks Der f extract 0.5 or 5 mg	5 × 25 μg Der f extract i.n.	(Shima K *et al.*) [37]
HDM	2 × 0.5 DU Der f extract + Alhydrogel i.p. or 4 × 1.5 DU Der f extract i.n.	SLIT 2 cycles 5 × 3 or 12 DU of Der f extract or 4 cycles: 3 × SLIT 12.6 DU extract	4 × 0.5 DU Der f extract i.n.	(Hagner S *et al.*) [38]
OVA	OVA+ Alum i.p.	2 × 0.2 mg OVA or SHAS-OVA s.c.	n/a	(Garbani M *et al.*) [41]
OVA	2 × 10 μg OVA + 1 mg Alum i.p.	3 × OVA+VD3 and CpG powder for 2 h on laser-treated skin, μEPIT or 8 × s.c.-treatments with 0.5, 1, 2, 4, 8, 16, 32, 50 μg OVA	3 × 0.500 μg OVA i.n.	(Kumar MN *et al.*) [44]
OVA	2 × 10 μg OVA + 3 mg Alum i.p.	Prophylactic or therapeutical 2 × PLG-OVA i.v.	3 × 20 min with 10 mg/ml OVA aerosol	(Smarr CB *et al.*) [46^▪▪^]
OVA	4 × 1 mg OVA + 20 μg cholera toxin o.g.	Oral 2 × 1 mg OVA, 2 × 5 mg OVA, 3 × 10 mg OVA, 2 × 25 mg OVA, 5 × 50 mg OVA +/– 10^9C. butyricum o.g.	n/a	(Shi Y *et al.*) [42^▪^]
OVA	4 × 100 μg OVA +/– 100 μg CpG e.c. left total of 14 days	2 × OVA or OVA+CpG e.c. left total of 8 days	2 × OVA e.c.	(Majewska-Szczepanik *et al.*) [45]
OVA	3 × 25 μg OVA + 1 mg Alum i.p.	6 × 10 μg OVA and 10 μg FlaB i.n. or sublingual or 2 × 10 μg OVA and 1 μg FlaB i.l.	6 × OVA 100 μg i.n.	(Kim EH *et al.*) [49^▪^]
OVA or Peanut	100 μg OVA or 1 mg peanut extract on shaved skin	8 × 100 μg OVA e.c. or 8 weeks 1 mg OVA orally	Oral challenge with 10, 20 and 50 mg OVA every 30 min	(Tordesillas L *et al.*) [43^▪▪^]
rCul n 3, rCul n4, rCul n8 rCul n10	n/a	3 × 10 μg of rCul n 3, rCul n4, rCul n8 rCul n10 + 500 μg Alum or Alum+50 μg MPLA (Avantilipids) s.l. in iceland horses	n/a	(Jonsdottier S *et al.*) [51]

e.c., epicutaneous application; HDM, house dust mite; i.n., intranasal; i.p., intraperitoneal; i.v., intravenous; n/a, not applicable; o.g., oral gavage; OVA, ovalbumin; rCul, recombinant *Culicoides nubeculosus* allergens; s.c., subcutaneous; s.l., submandibular lymph node; t.a., topical application.
